# Effects of Silica Nanoparticles on Brain Biomarkers in TgAPP/PS1/tau Mice

**DOI:** 10.1002/alz70856_102108

**Published:** 2025-12-25

**Authors:** Ling Guo, Baolian Dong, Chao Song, Zhendan He, Hongjiang Zhang, Guilan Xia, Yungui Tu, Qiong Du, Yue Feng, Xueshan Xia

**Affiliations:** ^1^ Anning First People's Hospital Affiliated to Kunming University of Science and Technology, Kunming, Anning, Yunnan, China

## Abstract

**Background:**

To know if SiO2 Nanoparticles (SiNPs) can increase the risk of Alzheimer's disease (AD) or cause worse damage to AD brains, SiNPs were introduced into wild type or TgAPP/PS1/tau mice by intranasal instillation (INI) in parallel till 28d, and biomarkers of AD and their mRNA as well as some apoptosis related proteins were tested.

**Method:**

Brain cortex obtained at 28d with 1‐4% of SiNPs exposure via intranasal instillation to WT or TgAPP/PS1/tau mice were measured Alzheimer's biomarkers including Aβ, BACE1 and tau or the proteins related to apoptosis such as caspase‐3, cleaved caspase‐3 or Bax via semi‐quantified by Western Blotting while mRNA events in blood serum from the animals were measured by RT‐qPCR.

**Result:**

Mean amyloid β_1–42_ (Aβ_1–42_), BACE1 protein levels in the brain cortex either WT or TgAPP/PS1/tau mice treated with SiNPs were significantly higher than those with NS‐controls. However, The levels of mRNA of APP, PS1 and tau at 28d via INI were significantly higher than for those with NS‐controls only in WT mice, which showed a patton in a concengtration‐dependent manner. Caspase‐3 remained not much change while cleaved caspase‐3 showed obvious upregulation in both type of mouse brains even at 1% SiNPs (concentratioon) exposure. The upregulation of Bax showed more in WT than in TgAPP/PS1/tau mice.

**Conclusion:**

Intranasally administered SiNPs with AD or WT mice may directly go to brains faster, disrupt the integrity of the cerebral cotex and alter the expression of proteins involved in biomarkers of AD, apoptosis, autophagy, and the neuronal cell lesion, suggesting their potential high risk to AD even normal people with more neurotoxic effects. This work presented the evidence of SiO2 NPs‐induced AD neurotoxicity.

